# High-performance hybrid nanogenerator for self-powered wireless multi-sensing microsystems

**DOI:** 10.1038/s41378-023-00563-7

**Published:** 2023-07-21

**Authors:** Dan-Liang Wen, Peng Huang, Hai-Tao Deng, Xin-Ran Zhang, Yi-Lin Wang, Xiao-Sheng Zhang

**Affiliations:** School of Integrated Circuit Science and Engineering, University of Electronic Science and Technology of China, Chengdu, 611731 China

**Keywords:** Electrical and electronic engineering, Electronic devices

## Abstract

Wireless sensor network nodes are widely used in wearable devices, consumer electronics, and industrial electronics and are a crucial component of the Internet of Things (IoT). Recently, advanced power technology with sustainable energy supply and pollution-free characteristics has become a popular research focus. Herein, to realize an unattended and reliable power supply unit suitable for distributed IoT systems, we develop a high-performance triboelectric-electromagnetic hybrid nanogenerator (TEHNG) to harvest mechanical energy. The TEHNG achieves a high load power of 21.8 mW by implementing improvements of material optimization, configuration optimization and pyramid microstructure design. To realize a self-powered integrated microsystem, a power management module, energy storage module, sensing signal processing module, and microcontroller unit are integrated into the TEHNG. Furthermore, an all-in-one wireless multisensing microsystem comprising the TEHNG, the abovementioned integrated functional circuit and three sensors (temperature, pressure, and ultraviolet) is built. The milliwatt microsystem operates continuously with the TEHNG as the only power supply, achieving self-powered operations of sensing environmental variables and transmitting wireless data to a terminal in real time. This shows tremendous application potential in the IoT field.

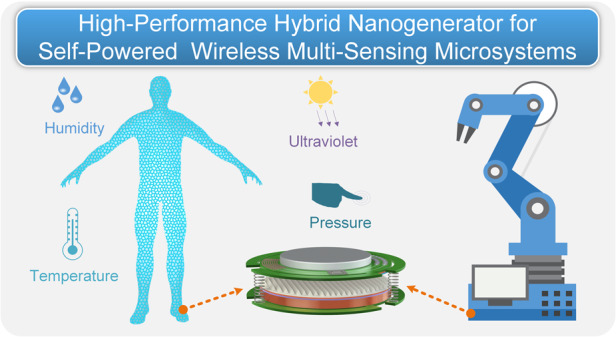

## Introduction

Wireless sensor network nodes that integrate power, diverse sensing, communication, and other functional components are essential components of the Internet of Things (IoT), which is developing rapidly with the gradual popularization of fifth generation mobile communication technology (5 G) and the continuous breakthrough of electronic information technology^1–3^. Conventional lithium batteries and nickel-zinc batteries, which are currently the most commonly used power supply units for industrial and wearable electronic devices, offer critical drawbacks such as frequent charging (or disposable devices), poor flexibility, and environmental pollution. Such equipment no longer satisfies the increasing requirements of portable power sources capable of advancing the rapid development of IoT. Therefore, the development of reliable portable power sources to overcome the above challenges is of great importance for further advancement and widespread applications of industrial electronics and wearable electronic devices, making this space an attractive research field^4–8^.

In recent years, an emerging technology for mechanical vibration energy harvesting based on the coupling of the triboelectric effect and electrostatic induction, namely, triboelectric nanogenerators (TENGs), have been proposed and have experienced vigorous development^9–14^. The basic theory of TENGs is introduced in detail in Fig. S1 in the Supporting Information file^15^. TENG has been proven to offer many advantages, such as excellent sustainability, high output performance, simple preparation process, and diverse material selection^16,17^. Therefore, it is a reliable and efficient approach to address the sustainable power supply issue of industrial and wearable electronic devices, and it has been widely applied to realize self-power sensors^18–22^, self-powered actuators^23–25^, self-powered microsystems^26–29^, and so on^30–33^. However, energy harvesters based on a single triboelectric mechanism present low conversion efficiency of mechanical energy. Thus, methods have been developed to improve it, such as introducing high-performance materials^34–36^, processing surfaces with microstructures^37^, studying/compositing energy conversion mechanisms^38,39^, and improving energy transfer efficiency^40–42^. Nevertheless, hardly any completely self-powered real-time continuous wireless multisensing microsystem have been demonstrated due to the insufficient load power capability of the energy harvester.

In this work, to realize a completely self-powered real-time continuous wireless multisensing microsystem, we develop a high-performance triboelectric-electromagnetic hybrid nanogenerator (TEHNG) based on a circular spring-cantilever structure. The optimized triboelectric pair of polyvinyl chloride (PVC) and polydimethylsiloxane (PDMS) was selected after experimental investigation. The configuration of the copper coil and magnet for the electromagnetic part was optimized by comparing magnets with different diameters. To collect the output energy of the TEHNG and continuously send sensing data, a combined power management module (PMM) circuit and energy storage circuit were integrated to efficiently store the obtained electricity. Moreover, a sensing signal processing circuit and a microcontroller unit (MCU) were implemented to process and transmit sensing signals. These four parts were integrated into the TEHNG to realize an all-in-one self-powered microsystem. As a result, the electricity obtained by the TEHNG can drive three sensors (temperature, pressure and ultraviolet (UV)) to work and simultaneously power the MCU to send sensing signals by internal Bluetooth, which is real-time rather than having a long period. The outcome is an all-in-one completely self-powered wireless multisensing microsystem (total power consumption 1.019 mW) that can continuously send data in real time by using TEHNG. This result presents great potential to replace lithium batteries and potentially introduce new developments in the field of wearable and industrial self-powered electronic devices.

## Results and discussion

### Structure of TEHNG

**Fig. 1:**
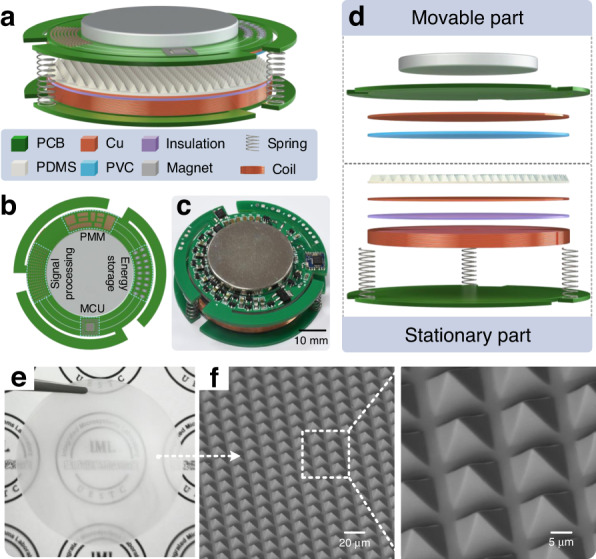
Triboelectric-electromagnetic hybrid nanogenerator (TEHNG) and integrated functional circuit for an all-in-one completely self-powered real-time multisensing microsystem. **a** The hybrid TEHNG is composed of a stationary part and a movable part. **b** The integrated functional circuit, including a power management module (PMM) circuit, an energy storage circuit, a microcontroller unit (MCU), and a sensing signal processing circuit, is integrated with the TEHNG. **c** The photograph of the fabricated TEHNG with the integrated functional circuit shows that its diameter is 60 mm and its height is 15 mm. **d** The exploded side view shows the detailed hierarchical structure of the developed TEHNG. The triboelectric part is made of an upper electrode, a polyvinyl chloride (PVC) triboelectric layer, a polydimethylsiloxane (PDMS) triboelectric layer, and a lower electrode. The electromagnetic part consists of a copper coil and a permanent magnet. The lower electrode of the triboelectric part and the coil of the electromagnetic part are isolated by a polyimide (PI) isolation layer. **e** Photograph of a 0.5 mm-thick microstructured PDMS film. **f** SEM images and high-magnification details reveal that the pyramid microstructures (13 μm in width and 4 μm in gap) were well transferred from the silicon template to PDMS, which can greatly increase the surface area of the PDMS film

Figure 1 shows schematic diagrams and photographs of the high-performance TEHNG. As shown in the three-dimensional (3D) schematic diagram in Fig. 1a, due to the strong rigidity of springs, a spring-cantilever structure was introduced to make the movable part more flexible. The configuration of a 10000-turn coil made of copper (50 mm in diameter and 5 mm in height) and a permanent magnet made of neodymium iron boron formed the electromagnetic part. The size of the magnet was optimized to a diameter of 35 mm and a height of 4 mm, which is analyzed in detail in Fig. S2 (Supporting Information). A PDMS film with a thickness of 0.5 mm and a PVC film with a thickness of 0.2 mm were selected as a triboelectric pair, which combined two copper electrodes to further form the triboelectric part. The selection process for the triboelectric pair is shown in Section electrical characteristics of the triboelectric part. To realize high integration, an integrated functional circuit composed of a PMM circuit, an energy storage circuit, an MCU, and a sensing signal processing circuit was processed on the upper PCB substrate, as shown in Fig. 1b, c. Among these components, the PMM was used to improve the energy transfer efficiency from TEHNG to the energy storage unit. An energy storage circuit consisting of several capacitors was implemented to store the harvested electrical energy. Moreover, the sensing data were obtained by the sensing signal processing circuit, and the function of the MCU was to control the sensing signal processing circuit to collect sensing data and send it to one or more terminals. Figure 1d is an exploded side view of the developed TEHNG, showing its detailed hierarchical structure.

To improve the output performance of the triboelectric part, a pyramid microstructure array was introduced to increase the surface area of the PDMS film. Figure 1e shows a photograph of a 0.5 mm-thick microstructured PDMS film. The PDMS film prepared from the silicon template had regular pyramid patterns, indicating that microstructures were well transferred from the silicon template to the PDMS film, as shown in the SEM image and magnified details in Fig. 1f. The improvement in the output performance of the triboelectric part by using microstructured PDMS is analyzed in detail in electrical characteristics of the triboelectric part.

### Working mechanism of TEHNG

**Fig. 2:**
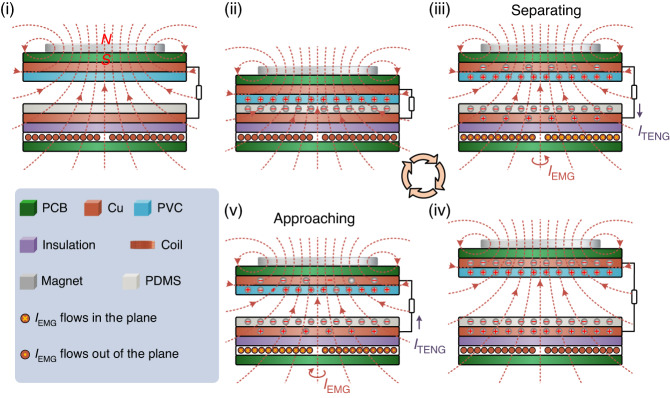
Schematic view of the working mechanism of the TEHNG for mechanical energy harvesting. (i)–(ii) When the movable part of the TEHNG is driven to contact the stationary part by an external mechanical force, equal amounts of positive and negative charges will be generated in the PVC film and PDMS film due to the triboelectrification effect. (ii)–(iii)–(iv) When the movable part separates from the stationary part after removing the external mechanical force, electrons flow from the lower electrode adjacent to the PDMS film to the upper electrode adjacent to the PVC film due to electrostatic induction. Meanwhile, owing to electromagnetic induction, an anti-clockwise current (top view) will be generated in the closed coil, which is caused by the decrease in upward magnetic flux in the separation process. (iv)–(v)–(ii) When the movable part of the TEHNG is driven to approach the stationary part again, electrons will flow back from the upper electrode to the lower electrode due to electrostatic induction. Meanwhile, the increase in upward magnetic flux will produce a clockwise current (top view) in the closed coil in the approaching process. Therefore, a cyclic mechanical input can simultaneously excite periodical triboelectric output and electromagnetic output

The working mechanism of the triboelectric part is based on the coupling of the triboelectrification effect and electrostatic induction. The working mechanism of the electromagnetic part is based on electromagnetic induction. Figure 2 demonstrates the process of the TEHNG generating triboelectric signals and electromagnetic signals. As shown in Fig. 2(i)–(ii), when an external mechanical force is applied to the movable part and makes it contact the stationary part, equivalent positive and negative charges are generated in the PVC film and PDMS film due to the triboelectrification effect. When this external mechanical force is removed and the movable part separates from the stationary part, electrons transfer from the lower electrode adjacent to the PDMS film to the upper electrode adjacent to the PVC film due to electrostatic induction, as shown in Fig. 2(ii)–(iii)–(iv). When the external mechanical force drives the movable part back into contact with the stationary part, electrons are transferred in the opposite direction as shown in Fig. 2(iv)–(v)–(ii). Therefore, a triboelectric signal is obtained in the external circuit. Simultaneously, when the movable part is driven to contact the stationary part, the upward magnetic flux at the coils increases, resulting in a clockwise electromagnetic current (top view) being produced in the coil because of the electromagnetic induction, as shown in Fig. 2(iv)–(v)–(ii). When the movable part separates from the stationary part, the upward magnetic flux at the coils decreases, leading to an anti-clockwise electromagnetic current (top view) in the coil, as shown in Fig. 2(ii)–(iii)–(iv). The electromagnetic signal and the triboelectric signal are generated simultaneously. If an external mechanical force drives the movable part to cyclically approach and separate from the stationary part, then periodic electromagnetic signals and triboelectric signals are obtained.

### Electrical characteristics of the triboelectric part

**Fig. 3:**
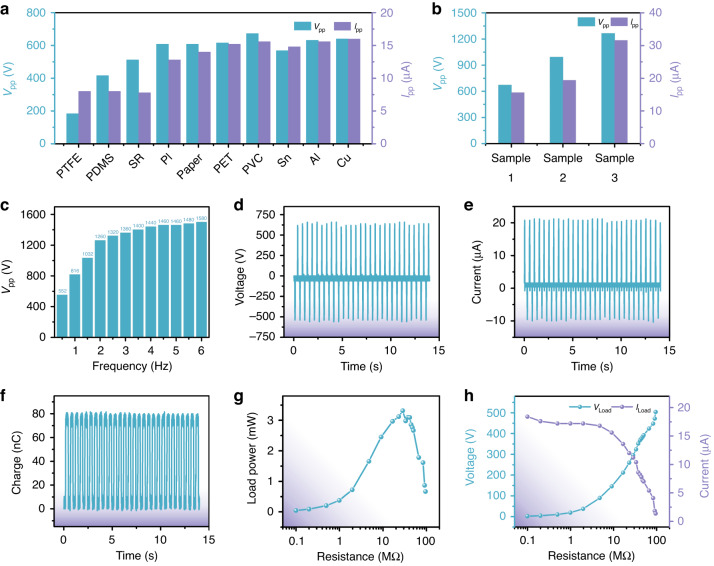
Systematic study of the electrical output characteristics of the triboelectric part. **a** The output peak-peak voltage (*V*_pp_) and peak-peak current (*I*_pp_) of the triboelectric part made of various materials with PDMS film indicate that the PVC film was the best choice. **b** Surface treatment with pyramid microstructures can increase the output performance of the triboelectric part, and a larger microstructure area (sample 1: without microstructures, sample 2: with half-area microstructures, sample 3: with full-area microstructures) led to more performance improvement. **c** The *V*_pp_ of the triboelectric part increased with increasing vibration frequency. **d**, **e** The average *V*_pp_ and *I*_pp_ under the 2 Hz vibration platform achieved ~1180.0 V and 30.4 μA, respectively. **f** The average amount of transferred charges in every working cycle of the triboelectric part achieved 79.8 nC. **g** The changing trend of the output power of the triboelectric part on the load under the 2 Hz vibration platform reveals that the maximum load output power reached 3.32 mW when the load resistance was 28.5 MΩ. **h** Dependence of the output voltage and current of the triboelectric part on the various load resistors

We conducted a series of experiments to optimize the output performance of the triboelectric system. PDMS comprising a mixed solution of base and curing agent is easily embedded with microstructures through casting. The PDMS film also presents high durability that is conducive to constructing a TENG and was thus selected as one of the triboelectric materials in this work. The other triboelectric material was selected by exploring different materials to form TENGs with PDMS and comparing their output performance. Figure 3a shows the peak-peak voltage (*V*_pp_) and peak-peak current (*I*_pp_) of TENGs composed of smooth PDMS films and varying materials, including poly tetra fluoroethylene (PTFE), PDMS, silicon rubber (SR), PI, paper, polyethylene terephthalate (PET), PVC, tin (Sn), aluminum (Al), and copper (Cu). The TENG comprising a smooth PDMS film and a PVC film showed the best output performance of ~672.0 V and 15.6 μA. This result is attributed to the native microstructures on the surface of the PVC film. Considering Fig. 3a and the above analysis, we conclude that PVC film is the best triboelectric material for building a TENG with PDMS. Furthermore, microstructure treatment was applied to enlarge the surface area of the PDMS triboelectric layer to improve the output performance of the triboelectric part. Figure 3b shows the output *V*_pp_ and *I*_pp_ of the fabricated TENGs with different areas of pyramid microstructures, including sample 1: without microstructures; sample 2: with half-area microstructures; and sample 3: with full-area microstructures. With the enlargement of the area of the pyramid microstructures, the output *V*_pp_ and *I*_pp_ showed a trend of stable increase, indicating that a PDMS film with full-area microstructures is more suitable for building the triboelectric part. The dependence of *V*_pp_ on frequency was studied by comparing the output *V*_pp_ of the triboelectric part at different vibration frequencies from 0.5 Hz to 6 Hz, as shown in Fig. 3c. When the vibration frequency increased from 0.5 Hz to 2 Hz, the *V*_pp_ showed a trend of rapid increase. When the vibration frequency increased from 2 Hz to 6 Hz, *V*_pp_ increased slowly. Considering that the frequency of human movement is usually <2 Hz, a systematic investigation of the output performance was conducted at 2 Hz. As shown in Fig. 3d, e, the average values of *V*_pp_ and *I*_pp_ reached 1180.0 V and 30.4 μA, respectively. The average amount of charge transferred in one working cycle of the triboelectric part was ~79.8 nC, as shown in Fig. 3f. Figure 3g shows the output performance of the triboelectric part to an external load with various resistances from 0.1 kΩ to 95.2 MΩ. We mention that the resistances used in the calculation of load output power were actual values, which are the parallel resistance values of the probe resistance and the resistances connected to the TENG. At a vibration frequency of 2 Hz, as the resistance increased from 0.1 KΩ to 28.5 MΩ, the load output power of the triboelectric part to the resistors had a rising trend from 0.04 mW to 3.32 mW. However, when the load resistance continued rising from 28.5 MΩ to 95.2 MΩ, the power output to the loads decreased from 3.32 mW to 0.66 mW. Therefore, the maximum output power of the triboelectric part to external resistors reached 3.32 mW when the load resistance was 28.5 MΩ. Figure 3h shows the detailed trends of load voltage (*V*_Load_) and load current (*I*_Load_) as the resistance of the external loads increased from 0.1 kΩ to 95.2 MΩ.

### Electrical characteristics of the electromagnetic part

**Fig. 4:**
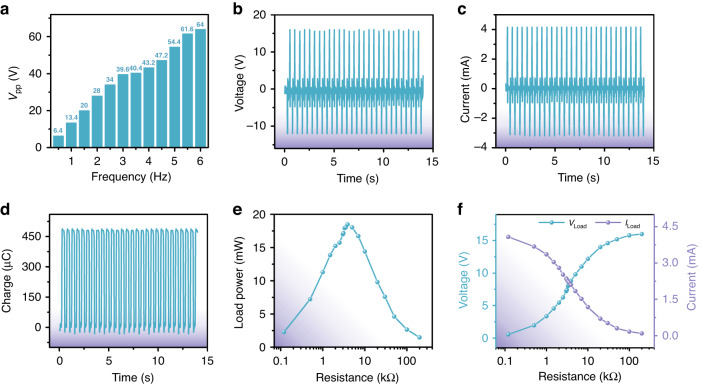
Electrical characterization of electromagnetic output by using a vibration platform. **a** The *V*_pp_ of the electromagnetic part increased with increasing driving frequency of the vibration platform. **b**, **c** The average *V*_pp_ and *I*_pp_ of the electromagnetic part driven by a 2 Hz vibration frequency achieved ~28.0 V and 7.4 mA, respectively. **d** The average amount of transferred charges in every working cycle of the electromagnetic part reached 480 μC. **e** Dependence of output power of the electromagnetic part on the various load resistors. The maximum load output power of 18.48 mW was obtained when the load resistance was 3.90 kΩ, which is consistent with the resistance of the coil. **f** Dependence of output voltage and current of the electromagnetic part on the various load resistors

Comprehensive measurements were conducted to systematically study the output performance of the electromagnetic part of the TEHNG. Figure 4a shows the investigation of the vibration frequency effect on the electromagnetic output. As the vibration frequency of the established vibration platform increased from 0.5 Hz to 6 Hz, the *V*_pp_ of the electromagnetic part gradually increased from 6.4 V to 64.0 V. Considering that the frequency of human movement is usually <2 Hz, we adopted the vibration frequency of 2 Hz to investigate the output characteristics of the electromagnetic part. Figure 4b, c exhibit the output voltage and output current of the electromagnetic part when the vibration frequency was 2 Hz. The average values of *V*_pp_ and *I*_pp_ achieved ~28.0 V and 7.4 mA, respectively. The average amount of charge transferred in one working cycle of the electromagnetic part reached ~480.0 μC, as shown in Fig. 4d. The output performance with respect to external loads was obtained by connecting the electromagnetic part to resistors with different values ranging from 0.1 kΩ to 199.6 kΩ, as shown in Fig. 4e. The resistances used to calculate the load output power were still the parallel resistances of the probe resistance and the resistances connected to the electromagnetic part. At 2 Hz, the output power of the electromagnetic part on the load resistors showed a rising trend from 2.28 mW to 18.48 mW as the load resistance increased from 0.1 kΩ to 3.8 kΩ. However, when the load resistance continuously increased from 3.8 kΩ to 199.6 kΩ, the output power of the electromagnetic part on the load resistors showed a decrease from 18.48 mW to 1.47 mW.

In other words, the maximum load output power of 18.48 mW can be obtained when the electromagnetic part of the TEHNG is connected to a resistor of 3.8 kΩ, which is almost the same as the internal resistance of the coil. Figure 4f demonstrates the dependence of *V*_Load_ and *I*_Load_ of the electromagnetic part on the load resistance from 0.1 kΩ to 199.6 kΩ.

### Electrical characteristics of TEHNG under foot stomping

**Fig. 5:**
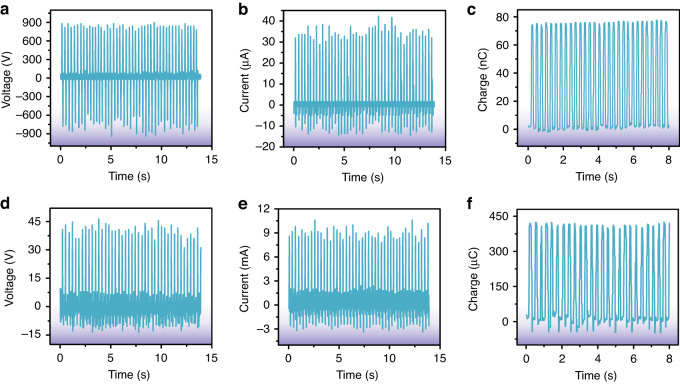
Electrical measurement of the TEHNG driven by foot stomping (~3 Hz). **a**, **b** The average *V*_pp_ and *I*_pp_ of the triboelectric part reached 1606.9 V and 48.5 μA under foot stomping, which were greatly enhanced compared with the average *V*_pp_ and *I*_pp_ of the triboelectric part driven by the 2 Hz vibration platform (1180.0 V and 30.4 μA). **c** The average amount of charges transferred in every working cycle of the triboelectric part was 76.2 nC. **d**, **e** The average *V*_pp_ and *I*_pp_ of the electromagnetic part significantly improved from 28.0 V and 7.4 mA (under 2 Hz vibration frequency) to 48.8 V and 12.8 mA (under foot stomping). **f** The average amount of transferred charges in every working cycle of the electromagnetic part driven by foot stomping was 419.2 μC

Furthermore, considering that the actual application scenarios are wearable energy harvesting, the output performance of the proposed TEHNG under wearable conditions was also studied. Figure 5 shows the output of the TEHNG as driven by foot stomping. The average *V*_pp_ and *I*_pp_ of the triboelectric part greatly increased from 1180.0 V and 30.4 μA under a 2 Hz vibration frequency to 1606.9 V and 48.5 μA under foot stomping (~3 Hz), as shown in Fig. 5a, b. We infer that foot stomping produced a greater contact force, which led to a larger contact area and a faster speed of approaching and separating. The average amount of charges transferred in one working cycle of the triboelectric part under foot stomping was 76.2 nC, as shown in Fig. 5c. On the other hand, the average *V*_pp_ and *I*_pp_ of the electromagnetic part reached 48.8 V and 12.8 mA (under foot stomping, ~3 Hz), which were much higher than those under the 2 Hz vibration platform (28.0 V and 7.4 mA), as shown in Fig. 5d, e. The average amount of charges transferred in one working cycle of the electromagnetic part under foot stomping reached 419.2 μC, as shown in Fig. 5f. Therefore, the proposed TEHNG demonstrates better output performance in wearable application scenarios, as proven by the combination of Fig. 5 and the above analysis.

### Stability and reliability measurement

**Fig. 6:**
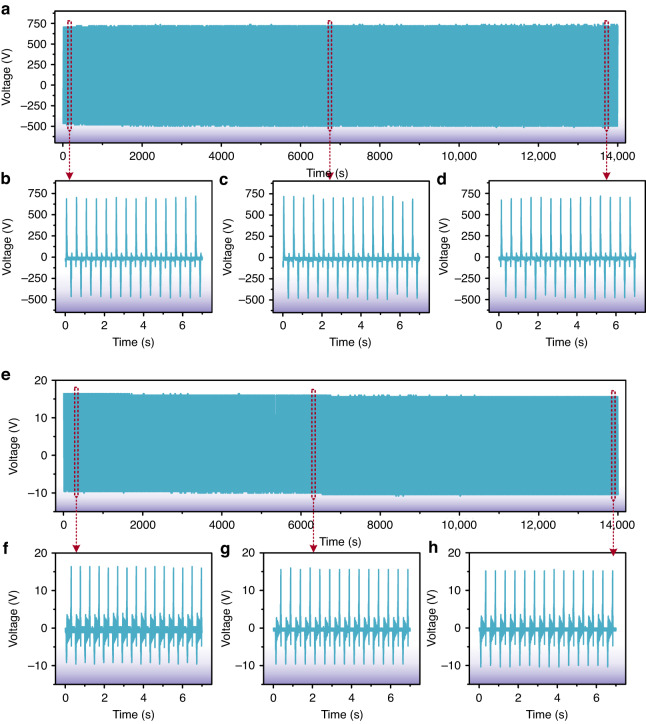
The output repeatability and stability of the developed TEHNG were evaluated by a 28000-time continuous operation experiment under a 2 Hz vibration platform. **a**–**d** The output voltage of the triboelectric part remained highly steady during the 28000-time continuous operation experiment. **e**–**h** The positive average output voltage of the electromagnetic part slightly decreased from 16.4 V to 16.0 V then to 15.6 V and then remained stable, while the negative average output voltage changed from −9.6 V to −10.0 V then to −10.4 V and then remained stable. Therefore, the *V*_pp_ of the electromagnetic part was completely consistent during the 28000-time continuous operation experiment. In summary, the fabricated TEHNG possesses remarkable output repeatability and stability, which are greatly beneficial for being a reliable power source

As a candidate for reliable power generation in wearable electronic devices, the TEHNG requires output stability and repeatability as two of its most crucial parameters for comprehensive evaluation. Thus, a 28000-time continuous operation experiment was implemented by using a 2 Hz vibration platform to evaluate the output stability and output repeatability of the TEHNG, as shown in Fig. 6. For the triboelectric part, the average positive and negative output voltages were always maintained at 720.0 V and −496.0 V during the entire repeatability experiment, as shown in the whole waveform in Fig. 6a and the enlarged details in Fig. 6b–d. For the electromagnetic part, the average positive output voltage slightly declined from 16.4 V to 16.0 V then to 15.6 V and then remained stable; simultaneously, the average negative output voltage slightly changed from −9.6 V to −10.0 V then to −10.4 V and then remained stable, as shown in the whole waveform in Fig. 6e and the enlarged details in Fig. 6f–h. Therefore, the *V*_pp_ of the electromagnetic part was always maintained at 26.0 V during the entire continuous repeatability experiment. In summary, Fig. 6 combined with the above analysis revealed the excellent output repeatability and stability of the developed TEHNG; thus, our device offers great potential as a reliable power supply unit for IoT devices.

## All-in-one self-powered multi-sensing microsystems

### Circuit design

**Fig. 7:**
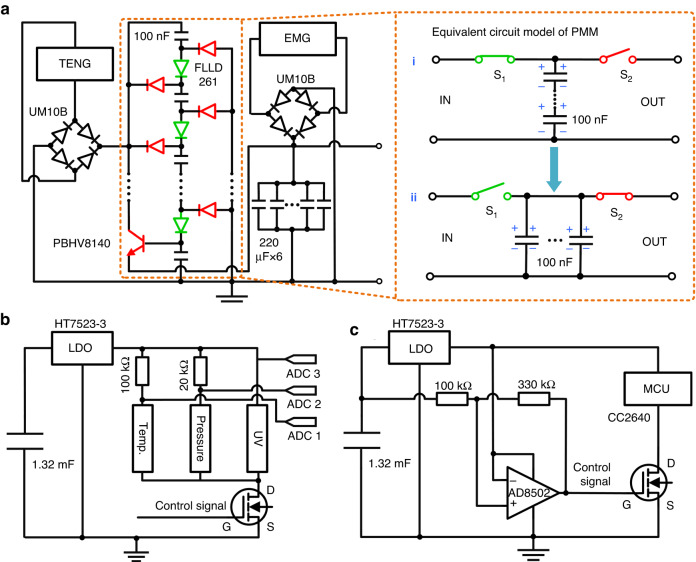
Circuit design for self-powered multisensing microsystems. **a** A PMM circuit based on a transitional capacitor array strategy is built to improve the energy transfer efficiency of the triboelectric part. **b** A signal processing circuit is designed to sample multiple sensing signals. **c** An automatic regulator circuit based on a bistable trigger is designed to control the working state of the MCU and signal processing circuit

To further demonstrate its feasibility and attractive application potential in self-powered industrial and wearable electronic devices, the TEHNG was used to drive an all-in-one multisensing microsystem that obtained environmental variables in real-time and sent these signals to one or more remote terminals. In addition to the TEHNG and environmental sensors, the all-in-one multisensing microsystem also included an integrated functional circuit, which comprised a PMM circuit, an energy storage circuit, an MCU, and a sensing signal processing circuit as shown in Fig. 7.

The output of a TENG has the common characteristic of high voltage but low current and total transported charge, resulting in a low energy transfer efficiency when powering electronics with relatively low impedances. However, there is currently no standard method to calculate the output impedance of the triboelectric part. Because the triboelectric part can be described by a variable capacitance model, a power management strategy based on a transitional capacitor array is an effective method to improve the energy transfer efficiency of TENGs^43^. We proposed such a transitional capacitor array circuit to reduce the output voltage and increase the output current of the TENG. The specific concept of our power management circuit is to charge the transition capacitors in series by a TENG to reduce the voltage on a single capacitor. The parallel capacitors then charge the energy storage capacitor to increase the output current. This circuit achieves impedance matching through series and parallel connection of transition capacitors to improve energy transmission efficiency. As shown in Fig. 7a, we studied the optimal transitional capacitor for the proposed triboelectric part by a comparative experiment, and the best capacitance was 100 nF. The working principle of the PMM of the triboelectric part is as follows. When the triboelectric part outputs the pulse voltage, it activates the green diodes, while the red diodes and triode turn off. Through these events, the transitional capacitors are connected in series (Fig. 7a–i), and the equivalent impedance is large. The generated electricity of the triboelectric part is then transferred to the series-connected transitional capacitors. When the output of the triboelectric part returns to 0, the green diodes turn off, while the red diodes and triodes turn on. Through these events, the transitional capacitors are connected in parallel (Fig. 7a–ii), and the equivalent impedance is small. The stored electricity in the parallel transitional capacitors is transferred to the energy storage unit. In summary, the triboelectric part achieves impedance matching through capacitor series-to-parallel conversion to reduce energy loss during transmission and improve energy transmission efficiency. Due to the low output impedance of the electromagnetic part, even if it is directly connected to the energy storage unit, a high energy transfer efficiency can be obtained. In our device, the electromagnetic output is stored in the energy storage capacitor only through a rectifier bridge. As a result, both the triboelectric part and electromagnetic part can effectively transmit energy to the energy storage unit through the power management circuit. Moreover, the energy storage circuit was a 1.32 mF capacitor constituted by six parallel 220 μF capacitors. An analog-to-digital converter (ADC) integrated in the MCU is combined with a resistance divider circuit to constitute the signal processing circuit for sampling multiple sensing signals, as shown in Fig. 7b. An automatic regulator circuit comprising a low dropout regulator (LDO), an MOS transistor, and a bistable flip-flop was designed to control and provide a stable power supply to the MCU unit, as shown in Fig. 7c. When the voltage of the energy storage circuit is charged to 3.3 V, the MOS turns on and the microsystem begins operation. When the voltage of the energy storage circuit is <2.3 V, the MOS turns off and the microsystem ceases operation.

**Fig. 8:**
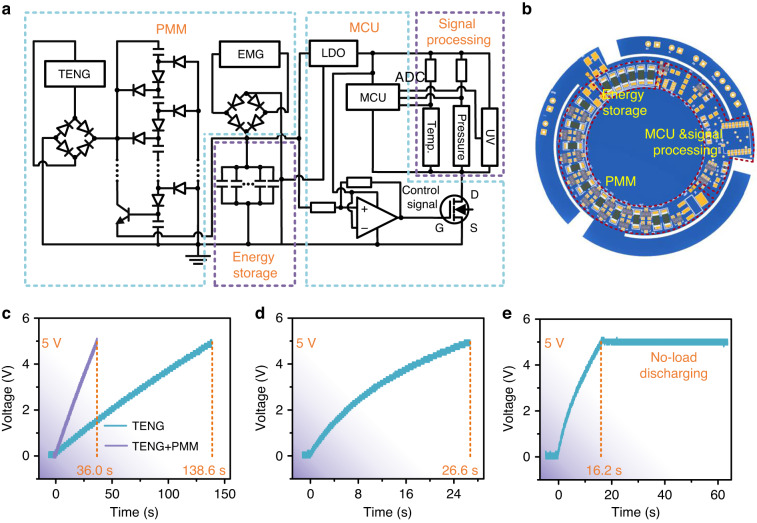
An integrated functional circuit was integrated with the TEHNG to form an all-in-one self-powered multisensing microsystem. **a**, **b** The integrated functional circuit was composed of a PMM circuit for improving energy transfer efficiency, an energy storage circuit for storing the obtained electricity, a signal processing circuit for sampling sensing signals, and an MCU unit for sending wireless data. **c** After using the PMM, the time of the triboelectric part charging a 10 μF capacitor to 5 V by a 2 Hz vibration platform decreased from 138.6 s to 36.0 s, indicating that the energy transfer efficiency of the triboelectric part was greatly improved. **d** The time of the TEHNG charging a 1.32 mF capacitor to 5 V under a 2 Hz vibration platform was 26.6 s. **e** The TEHNG driven by 2 Hz foot stomping can charge a 1.32 mF capacitor to 5 V within 16.2 s, showing a strong power supply capability. The stored electricity in capacitors hardly leaked without load

Furthermore, the integrated functional circuit composed of a PMM, an energy storage circuit, a signal processing circuit, a MCU and an automatic regulator circuit was first integrated into the TEHNG, as shown in Fig. 8a, b. Figure 8c shows the comparison of charging a 10 μF capacitor by the TEHNG with and without the PMM. Driven by the 2 Hz vibration platform, the TEHNG charging a 10 μF capacitor to 5 V took 36 s, which was shortened by ~74% compared with 138.6 s without the PMM, demonstrating that the energy transfer efficiency of the triboelectric part was greatly improved. To evaluate the power supply capacity of the TEHNG with PMM, it was implemented to charge a 1.32 mF capacitor to 5 V under a 2 Hz vibration platform and 2 Hz foot stomping. As shown in Fig. 8d, e, the charging times under the 2 Hz vibration platform and 2 Hz foot stomping were 26.6 s and 16.2 s, respectively, which showed a strong power supply capability. From Fig. 8e, we can conclude that the stored electricity in the 1.32 mF capacitor hardly leaked without load. Moreover, the comparison of charging a 1.32 mF capacitor by the TEHNG under different human movements is shown in Fig. S3.

### Microsystem integration

**Fig. 9:**
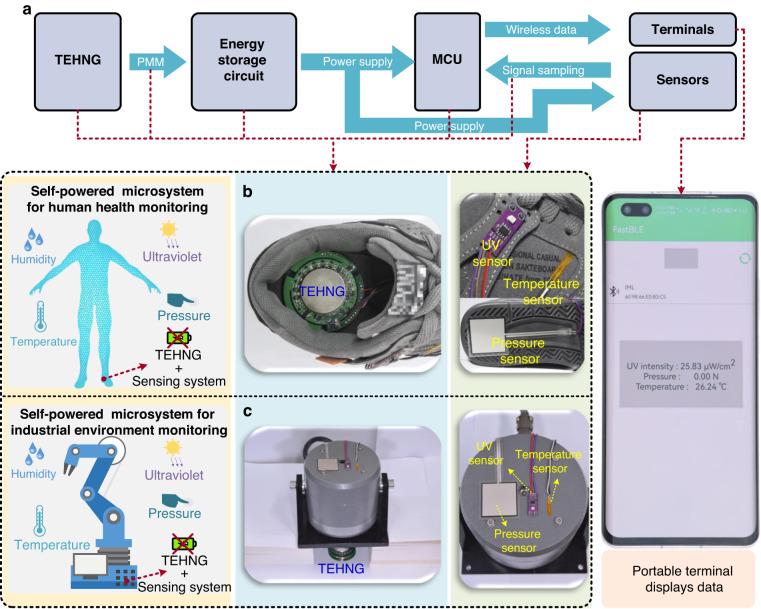
The proposed high-performance TEHNG with an integrated functional circuit has attractive potential to power industrial and wearable electronic devices, i.e., forming all-in-one completely self-powered microsystems. **a** Based on the triboelectric effect and the electromagnetic effect, the TEHNG converted mechanical energy into electrical energy, which was stored in an energy storage circuit through a high-efficiency PMM circuit. The stored electrical energy was used to power an MCU and multiple sensors at the same time without any other power source. The continuous sensing signals were sampled by the combination of an MCU and a signal processing circuit. Then, the sampled data were sent to one or more terminals by the internal Bluetooth of MCU and displayed. **b**, **c** The TEHNG was packaged inside a shoe and under a vibration platform. Commercial sensors, including temperature sensors, pressure sensors and ultraviolet (UV) sensors, were distributed around the shoe and on the vibration platform. A mobile phone was used to receive the wireless sensing data and display the environmental variables

Figure 9a shows the system diagram of the all-in-one self-powered multisensing microsystem. First, based on the triboelectric effect and the electromagnetic effect, the TEHNG harvested the mechanical energy of industrial machines or human movements and generated electricity, which was stored in an energy storage circuit through the PMM circuit. Then, the stored electricity was applied to power multiple sensors to obtain environmental variables. Simultaneously, the MCU was driven by the stored electricity to sample the sensing signals through a signal processing circuit. Finally, the sampled sensing signals were sent to one or more terminals by the internal Bluetooth transmitter of the MCU. In other words, the all-in-one multisensing microsystem can detect environmental variables, such as temperature, pressure, humidity, and UV, and send the sensor data without any additional power source, as shown in Fig. 9b, c. As a wearable electronic device, the TEHNG with an integrated circuit was packaged inside a shoe, and commercial sensors, including a temperature sensor, a pressure sensor, and a UV sensor, were distributed on the upper and bottom of the shoe. As an industrial electronic device, the TEHNG was placed under a vibration platform to harvest energy, and sensors were distributed on the machine to monitor the machine and the environment. Moreover, the wireless sensing data were obtained by a mobile phone and displayed after analysis. We note that the Bluetooth transmitter sent the sensor data via the broadcast function. Therefore, increasing the number of terminals would not increase the device’s power consumption.

**Fig. 10:**
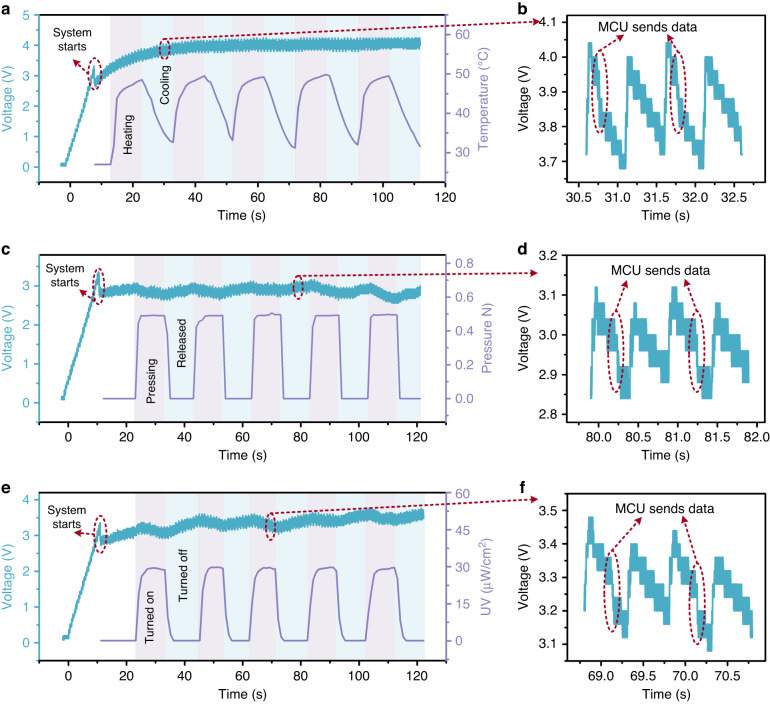
The MCU and sensors can continuously work when the TEHNG (2 Hz) is the only power source. In the experiment, the three sensors were simultaneously connected to the microsystem but were tested separately. **a** The voltage (green) of the energy storage circuit and the temperature sensing signal (purple) when the other two sensors were static. When the voltage of the energy storage circuit reached 3.3 V, the multisensing microsystem started. Then, the temperature sensor was cyclically placed on a heating plate at 50 °C for 10 s and removed for cooling for 10 s. Meanwhile, the MCU sent the temperature signal to a terminal at 1 Hz. **b** The operation of the MCU sending data increased the overall power consumption, resulting in a rapid drop in the stored voltage. **c**, **d** The voltage (green) of the energy storage circuit and the pressure sensing signal (purple) when a 50 g weight was cyclically applied to the pressure sensor for 10 s and released for 10 s. **e**, **f** The voltage (green) of the energy storage circuit and the UV sensing signal (purple) when a 30 μW/cm^2^ UV light was cyclically irradiated on the UV sensor for 10 s and turned off for 10 s. Among the sensors used, the temperature sensor and pressure sensor have the lowest and highest power consumption, respectively; thus, the corresponding final voltages were the largest and smallest, respectively. Moreover, to verify the feasibility of the all-in-one self-powered multisensing microsystem in practical applications, a realistic test of a 15-min walk was carried out. As a result, the microsystem can work continuously, as shown in Fig. S7 and Video S2 in the Supporting Information file. Additionally, the developed TEHNG can be integrated with a rechargeable battery to form a power source with self-charging and long-endurance characteristics for wider application scenarios

Figure 10 demonstrates the working states of the sensors and the corresponding voltage waveforms of the energy storage circuit. The three commercial sensors and MCU were simultaneously connected to the system. When the voltage of the energy storage circuit reached 3.3 V, the microsystem was controlled to start by the automatic regulator circuit. At a TEHNG working frequency of 2 Hz, the charging process typically took 8–10 s before the microsystem powered on. Then, we sequentially changed the input environmental variables around the sensors and recorded the waveform changes of the sensors and energy storage circuit. The temperature sensor was cyclically placed on a 50 °C heating plate for 10 s and removed for cooling for 10 s. The resulting temperature change of the temperature sensor is shown as the purple waveform in Fig. 10a. Notably, the temperature did not reach 50 °C and did not recover to room temperature due to the slow response speed of the temperature sensor used. The voltage of the energy storage circuit is shown as the green waveform in Fig. 10a. The MCU sent the temperature data to a mobile phone at a sending frequency of 1 Hz. Because sending data instantaneously increased the whole power consumption of the MCU (from 660 μW to 15 mW), the voltage of the energy storage circuit presented an instantaneous drop, as shown in Fig. 10b. The operation of the temperature sensor driven by the TEHNG is shown in Fig. S4. A 50 g weight was cyclically applied to the pressure sensor for 10 s and removed for 10 s, and the temperature sensor and UV sensor were static. The resistance of the pressure sensor greatly decreased as the pressure increased, resulting in a large increase in power consumption (from 15 μW to 196 μW), and the power consumption of the pressure sensor used was much larger than that of the temperature sensor (26 μW at room temperature and 35 μW at 50 °C). Therefore, the voltage of the energy storage circuit slightly decreased when the 50 g weight was placed on the pressure sensor and subsequently increased when the weight was removed from the pressure sensor, as shown by the green waveform in Fig. 10c. The pressure sensing signal is shown as the purple waveform in Fig. 10c. Figure 10d shows the detailed voltage waveform when the MCU sent data. Similarly, a 30 μW/cm^2^ UV light was cyclically irradiated on the UV sensor for 10 s and turned off for 10 s, and the temperature sensor and pressure sensor were static. Figure 10e, f show the voltage of the energy storage circuit and the UV sensing signal. The static and working power consumptions of the UV sensor used are ~95 μW and 172 μW, respectively. The sensing properties of the pressure sensor and UV sensor powered by TEHNG are shown in Figs. S5, S6, respectively. The detailed process of TEHNG powering the all-in-one wireless multisensing microsystem is exhibited in Supplementary Video S1. We note that in the video, to facilitate the display of the whole system and voltage waveform change, knocking was used instead of foot stomping to drive the TEHNG.

## Conclusions

Here, to address the sustainable power supply issue of wearable electronic devices, we developed a high-performance triboelectric-electromagnetic hybrid nanogenerator (TEHNG) to harvest biomechanical energy from human movements and generate electricity. A circular spring-cantilever structure was implemented to construct the TEHNG and realize some flexibility. The triboelectric part comprised an optimized triboelectric pair of materials including PVC and PDMS films. Pyramid array-shaped microstructures (13 μm in width) were embedded into the PDMS film to enhance the output performance. The electromagnetic part was composed of a 10000-turn coil and a size-optimized magnet. the resulting TEHNG achieved a maximum load output power of 21.8 mW, and the output presented remarkable stability during a 28000-time continuous repeatability experiment. To realize an all-in-one self-powered wireless microsystem, an integrated functional circuit comprising a PMM circuit for improving energy transfer efficiency, an energy storage circuit for storing the obtained electricity, a sensing signal processing circuit for sampling sensing signals, and an MCU for sending wireless data, was integrated in a device with the TEHNG. Finally, the TEHNG was successfully demonstrated to drive the MCU while sending wireless sensing data to a mobile phone at 1 Hz and continuously operating three environmental sensors in real time. In summary, an all-in-one completely self-powered wireless multisensing microsystem (1.019 mW) that can continuously send data to a terminal was achieved by using the TEHNG as the only power source. This TEHNG offers potential to replace lithium batteries in some specific scenarios and can greatly advance the development of self-powered wearable electronics.

## Experimental section

### Fabrication of TEHNG

The triboelectric part and electromagnetic part of the developed high-performance TEHNG are based on the contact-separation mode; therefore, the device is divided into a movable part and a stationary part in structural design, and a spring-cantilever structure was applied to connect the two parts in this work. The fabrication of the TEHNG is as follows. Two printed circuit boards (PCBs), each of which was processed with three cantilevers, were prepared as circles with a diameter of 60 mm serving as the substrate of the movable part and stationary part. A 10000-turn coil with a diameter of 50 mm and a height of 5 mm was fixed on the lower PCB substrate by tape. It should be noted that the surface of the coil and the lower PCB substrate has a layer of insulating material. A permanent magnet with a diameter of 35 mm and a height of 4 mm was fixed on the upper PCB substrate. The coil and the magnet formed the electromagnetic part. An insulating layer made of polyimide tape (PI) was cut into circles (50 mm in diameter) and attached to the coil before fabricating the triboelectric part for better insulation. Then, a circular copper film (i.e., triboelectric lower electrode) and a PDMS film (i.e., lower triboelectric layer), the diameters of which were 50 mm, were sequentially attached to the stationary part by insulating tape and conductive tape, respectively. Moreover, to improve the output performance of the triboelectric part, the PDMS film was processed with pyramid microstructures (13 μm in width and 4 μm in gap) from a silicon template through the inverted mold method. In brief, liquid PDMS was dispensed to the silicon template and cured in an oven at 70 °C for 1 h after being vacuumed. The triboelectric upper copper electrode was fabricated beneath the upper PCB substrate by the PCB etching process, and the upper triboelectric layer was fixed beneath the triboelectric upper electrode; thus, the triboelectric part was obtained. The lower electrode of the triboelectric part and the two ports of the coil were connected to the upper PCB substrate through wires. In addition, the integrated functional circuit was soldered to the upper PCB substrate. Finally, the ends of springs were adhered to the upper and lower PCB substrates to connect the movable part and stationary part.

### Tests and measurements

Field emission scanning electron microscopy (SEM, JSM-7600F, JEOL Ltd.) was used to characterize the surface morphologies of the smooth PDMS and the microstructured PDMS. To measure the output performance of the fabricated TEHNG, a vibration platform and a test platform were set up. The vibration platform was composed of a signal generator (33250 A, Agilent Technologies Inc.), an amplifier (YE5872A, Sinocera Piezotronics Inc.), and a shaker (JZK-10, Sinocera Piezotronics Inc.), which was applied to supply an external force with programmable frequency and magnitude to the TEHNG. The test platform contained a low-noise current preamplifier (SR570, Stanford Research Systems) and a digital oscilloscope (DS2302A, RIGOL Technology Co., Ltd.) with a 100 MΩ probe.

To further verify the output performance of the TEHNG and prove the feasibility of powering an all-in-one microsystem, an integrated functional circuit, including a PMM circuit, an energy storage circuit, an MCU (RF-BM-4044B4, Shenzhen RF-star Technology Co., Ltd.), and a sensing signal processing circuit, was integrated with the TEHNG, while three commercial sensors, including a temperature sensor (B3950, Shenzhen Yuxin Electronic Technology Co., Ltd.), a pressure sensor (IMS-S40A, Suzhou Nadu New Material Co., Ltd.), and a UV sensor (S12SD, Genicom) were connected to the TEHNG. In the measurement, the three sensors were connected to the system at the same time, but they were tested separately. The sensing data from sensors were continuously sent to a terminal, such as a mobile phone, by the internal Bluetooth in MCU. The terminal displayed the corresponding input environmental variables after parsing the sensing data.

## Supplementary information

Supplemental Material

Video S1

Video S2

## References

[1] Xing L (2020). Reliability in internet of things: current status and future perspectives. IEEE Internet Things J.

[2] Li S, Xu LD, Zhao S (2015). The internet of things: a survey. Inf. Syst. Front..

[3] Aun NFM (2017). Revolutionizing wearables for 5G: 5G technologies: recent developments and future perspectives for wearable devices and antennas. IEEE Microw. Mag..

[4] Xu C, Song Y, Han M, Zhang H (2021). Portable and wearable self-powered systems based on emerging energy harvesting technology. Microsyst. Nanoeng..

[5] Jiang D (2020). A leaf-shaped triboelectric nanogenerator for multiple ambient mechanical energy harvesting. IEEE Trans. Power Electron.

[6] Wen DL (2020). Wearable multi-sensing double-chain thermoelectric generator. Microsyst. Nanoeng..

[7] Song Y (2019). High-efficiency self-charging smart bracelet for portable electronics. Nano Energy.

[8] Ahmadi MH (2018). Renewable energy harvesting with the application of nanotechnology: a review. Int. J. Energy Res..

[9] Fan FR, Tian ZQ, Wang ZL (2012). Flexible triboelectric generator. Nano Energy.

[10] Li Y (2020). Electron transfer mechanism of graphene/Cu heterostructure for improving the stability of triboelectric nanogenerators. Nano Energy.

[11] Guo H (2015). A triboelectric generator based on checker-like interdigital electrodes with a sandwiched PET thin film for harvesting sliding energy in all directions. Adv. Energy Mater..

[12] Chen H, Song Y, Cheng X, Zhang H (2019). Self-powered electronic skin based on the triboelectric generator. Nano Energy.

[13] Yang H (2022). High-sensitive and ultra-wide spectrum multifunctional triboelectric acoustic sensor for broad scenario applications. Nano Energy.

[14] Yang H (2020). Polydirectional microvibration energy collection for self-powered multifunctional systems based on hybridized nanogenerators. ACS Nano.

[15] Niu S (2013). Theoretical study of contact-mode triboelectric nanogenerators as an effective power source. Energy Environ. Sci..

[16] Zou H (2019). Quantifying the triboelectric series. Nat. Commun..

[17] Jiang W (2018). Fully bioabsorbable natural-materials-based triboelectric nanogenerators. Adv. Mater..

[18] Yuan Z (2022). Integrated real-time pneumatic monitoring system with triboelectric linear displacement sensor. IEEE Trans. Ind. Electron..

[19] Wen DL (2019). Printed silk-fibroin-based triboelectric nanogenerators for multi-functional wearable sensing. Nano Energy.

[20] Moon JH (2021). Self-powered inertial sensor based on carbon nanotube yarn. IEEE Trans. Ind. Electron..

[21] Song Y, Wang N, Hu C, Wang ZL, Yang Y (2021). Soft triboelectric nanogenerators for mechanical energy scavenging and self-powered sensors. Nano Energy.

[22] Wu H, Shi Q, Wang F, Thean AVY, Lee C (2018). Self-powered cursor using a triboelectric mechanism. small methods.

[23] Chen X (2017). Fluid eddy induced piezo-promoted photodegradation of organic dye pollutants in wastewater on ZnO nanorod arrays/3D Ni foam. Mater. Today.

[24] Li Z (2016). Triboelectrification-enabled self-powered detection and removal of heavy metal ions in wastewater. Adv. Mater..

[25] Han K (2020). Wind-driven radial-engine-shaped triboelectric nanogenerators for self-powered absorption and degradation of NOX. ACS Nano.

[26] Zhang XS (2018). All-in-one self-powered flexible microsystems based on triboelectric nanogenerators. Nano Energy.

[27] Shi Q, Sun Z, Zhang Z, Lee C (2021). Triboelectric nanogenerators and hybridized systems for enabling next-generation IoT applications. Reserch.

[28] Yong S (2021). Auto-switching self-powered system for efficient broad-band wind energy harvesting based on dual-rotation shaft triboelectric nanogenerator. Adv. Energy Mater..

[29] Zhang X (2021). Harvesting multidirectional breeze energy and self‐powered intelligent fire detection systems based on triboelectric nanogenerator and fluid‐dynamic modeling. Adv. Funct. Mater..

[30] Bu T (2020). Nanoscale triboelectrification gated transistor. Nat. Commun..

[31] Wen F (2020). Battery-free short-range self-powered wireless sensor network (SS-WSN) using TENG based direct sensory transmission (TDST) mechanism. Nano Energy.

[32] Chen C (2020). Micro triboelectric ultrasonic device for acoustic energy transfer and signal communication. Nat. Commun..

[33] Chen X (2016). Stimulating acrylic elastomers by a triboelectric nanogenerator-toward self-powered electronic skin and artificial muscle. Adv. Funct. Mater..

[34] Chen A, Zhang C, Zhu G, Wang ZL (2020). Polymer materials for high-performance triboelectric nanogenerators. Adv. Sci..

[35] Zhang L (2020). Cellulose II aerogel-based triboelectric nanogenerator. Adv. Funct. Mater..

[36] Wen DL (2021). Recent progress in silk fibroin-based flexible electronics. Microsyst. Nanoeng..

[37] Zhang XS, Han MD, Meng B, Zhang HX (2015). High performance triboelectric nanogenerators based on large-scale mass-fabrication technologies. Nano Energy.

[38] Liu H, Fu H, Sun L, Lee C, Yeatman EM (2021). Hybrid energy harvesting technology: From materials, structural design, system integration to applications. Renew. Sust. Energ. Rev..

[39] Gao L (2019). Enhancing the output performance of triboelectric nanogenerator via grating-electrode-enabled surface plasmon excitation. Adv. Energy Mater..

[40] Hu T, Wang H, Harmon W, Bamgboje D, Wang ZL (2022). Current progress on power management systems for triboelectric nanogenerators. IEEE Trans. Power Electron.

[41] Yang J (2018). Managing and optimizing the output performances of a triboelectric nanogenerator by a self-powered electrostatic vibrator switch. Nano Energy.

[42] Li X, Sun Y (2022). An SSHI rectifier for triboelectric energy harvesting. IEEE Trans. Power Electron.

[43] Chen YL (2019). Self-powered smart active RFID tag integrated with wearable hybrid nanogenerator. Nano Energy.

