# Supplementary material for: High-performance hybrid nanogenerator for self-powered wireless multi-sensing microsystems
Source: Microsyst Nanoeng. 2023 Jul 21;9:94. doi: 10.1038/s41378-023-00563-7 (PMC10359314; doi:10.1038/s41378-023-00563-7)
Supplement: Supplementary file 1 — Supplemental Material [file 41378_2023_563_MOESM1_ESM.docx]

Supporting information

High-Performance Hybrid Nanogenerator for Self-Powered Wireless Multi-Sensing Microsystems

Dan-Liang Wen^1,#^, Peng Huang^1,#^, Hai-Tao Deng^1^, Xin-Ran Zhang^1^, Yi-Lin Wang^1^, Xiao-Sheng Zhang^1,*^

^1^School of Integrated Circuit Science and Engineering, University of Electronic Science and Technology of China, Chengdu 611731, China

^#^These authors contribute equally to this work.

*Corresponding Author: [zhangxs@uestc.edu.cn](mailto:zhangxs@uestc.edu.cn) (XS Zhang)


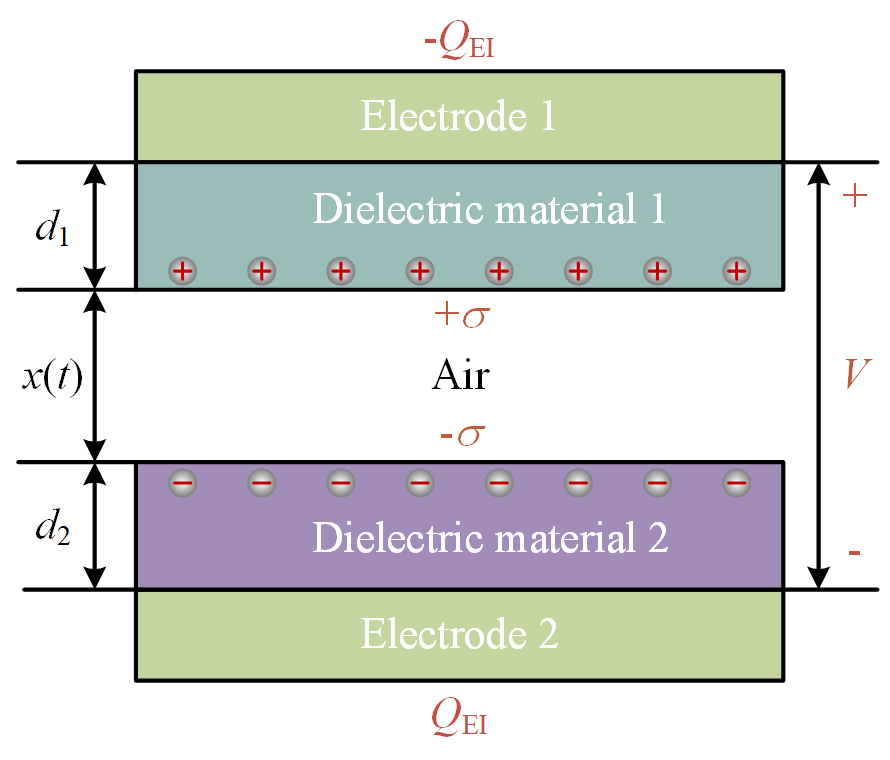


**Figure S1**. The working mechanism of triboelectric nanogenerator (TENG) is based on the coupling of the triboelectric effect and electrostatic induction. Triboelectric effect refers to the phenomenon that when two different objects get into contact with each other, one object will lose electrons and the other object will obtain electrons due to the difference in the electron binding capacity of nuclei.

The basic working principle of TENG is as follows [S1]. When the two dielectric materials are driven by external force to get into contact with each other, the electron transfer between the two materials will be generated due to the triboelectric effect. Positive and negative charges in equal amount will be generated on the surface of the two materials, and the amount of charge is $Q_{\text{TE}}$. When the two materials are separated, due to electrostatic induction, the electrode adjacent to the dielectric material will induce charges with the opposite polarity, the amount of induced charge is $Q_{\text{EI}}$.

Ideally, the expression of friction transferred charge $Q_{\text{TE}}$ is as follows:

$$Q_{\text{TE}}=S\sigma_{\text{TE}}$$

Where *S* is the area of the two dielectric materials, and $\sigma_{\text{TE}}$ is the surface charge density generated by the two materials during friction.

The calculation formula of potential difference between electrodes is as follows:

$$V\text{=}E_{1}\text{d}_{1}+E_{2}d_{2}+E_{\text{air}}x(t)$$

The electric field strength of the air between the dielectric materials is as follows:

$$E_{\text{air}}\text{=}\frac{-\frac{Q_{\text{EI}}}{S}+\sigma_{\text{TE}}}{\varepsilon_{0}}$$

The internal electric field strength of dielectric material 1 is as follows:

$$E_{1}\text{=}-\frac{Q_{\text{EI}}}{S\varepsilon_{0}\varepsilon_{\text{r}1}}$$

The internal electric field strength of dielectric material 2 is as follows:

$$E_{2}\text{=}-\frac{Q_{\text{EI}}}{S\varepsilon_{0}\varepsilon_{\text{r2}}}$$

Thus, the potential difference between electrodes is transformed as follows:

Where $\varepsilon_{0}$ represents the dielectric constant of air, and represent the dielectric constants of the two triboelectric materials, respectively, $d_{1}$ and $d_{2}$ represent the thicknesses of the two triboelectric materials, respectively, and $x(t)$ is the distance between the two dielectric materials.


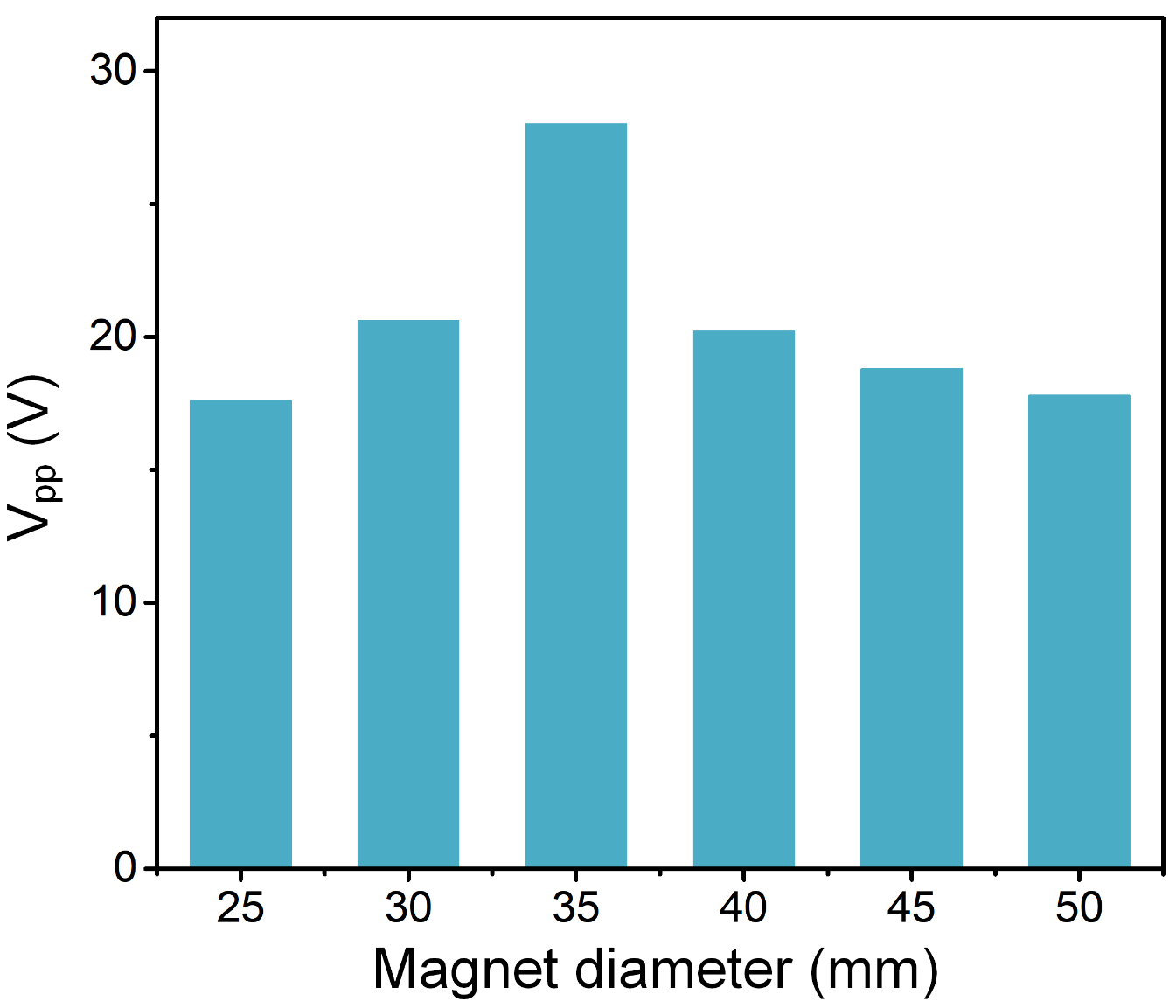


**Figure S2**. The effect of magnet diameter on the output performance of electromagnetic part of triboelectric-electromagnetic hybrid nanogenerator (TEHNG). The diameter of coil we used to construct electromagnetic part in this work was 50 mm. However, a magnet with the same diameter as the coil may not be the best choice because the magnetic field strength at the edge is higher than that at the center for circular magnets. Therefore, we compared the output performance of different electromagnetic combinations of a coil with a diameter of 50 mm and magnets with various diameters from 25 mm to 50 mm. As a result, the combination of a coil with a diameter of 50 mm and a magnet with a diameter of 35 mm presented the highest peak-peak voltage (*V*_pp_) of 28 V. Moreover, the magnetic field strength of the magnet is positively correlated with the thickness. To simultaneously ensure high output performance and light weight of TEHNG, the magnet thickness was set to 4 mm.


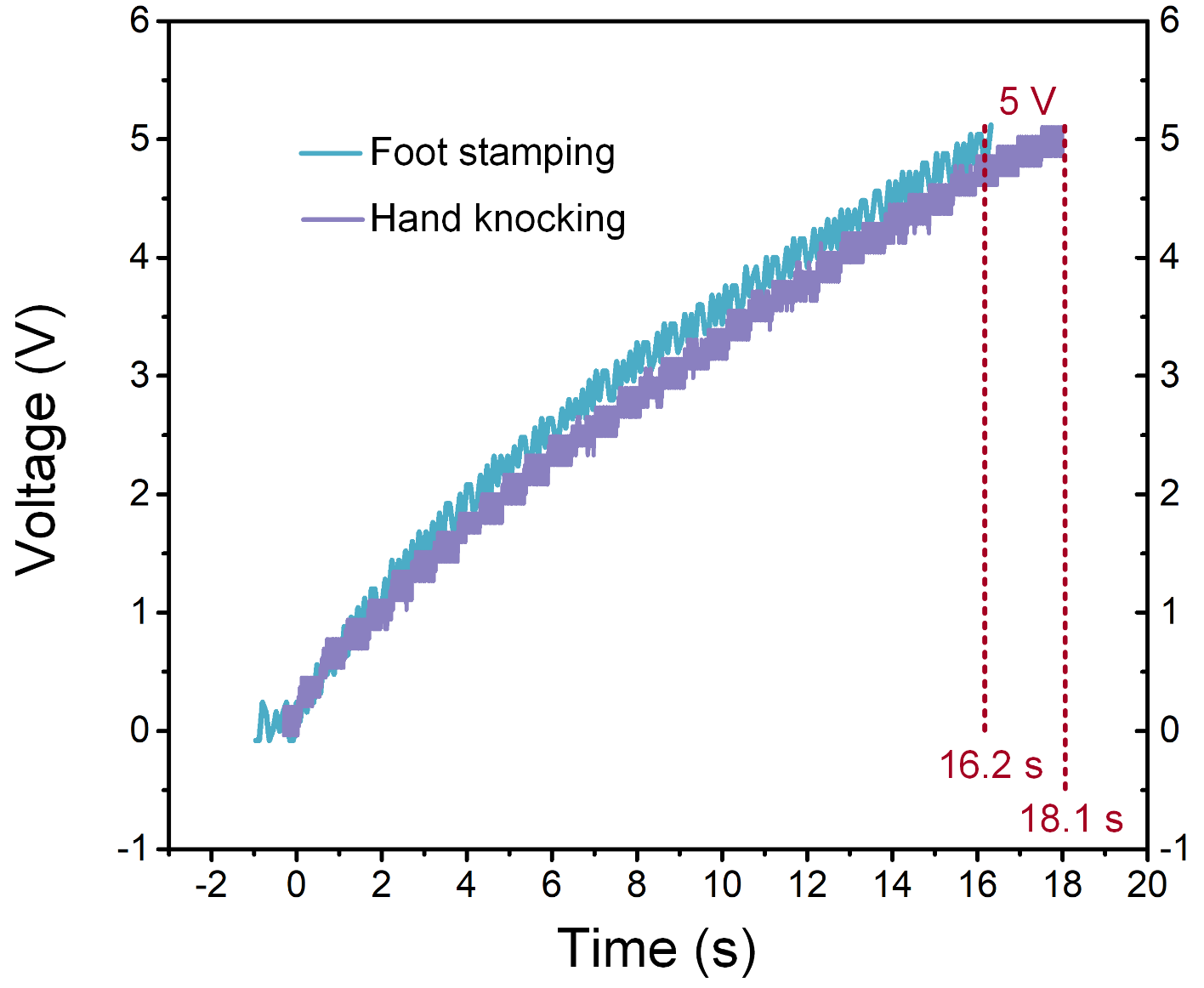


**Figure S3**. The comparison of charging a 1.32 mF capacitor by the TEHNG under foot stamping and hand knocking. The time of 1.32 mF capacitor charged to 5 V by the TEHNG under 2 Hz foot stamping and 2 Hz hand knocking were 16.2 s and 18.1 s. Different human movements generate different forces and different accelerations, resulting in different output characteristics.


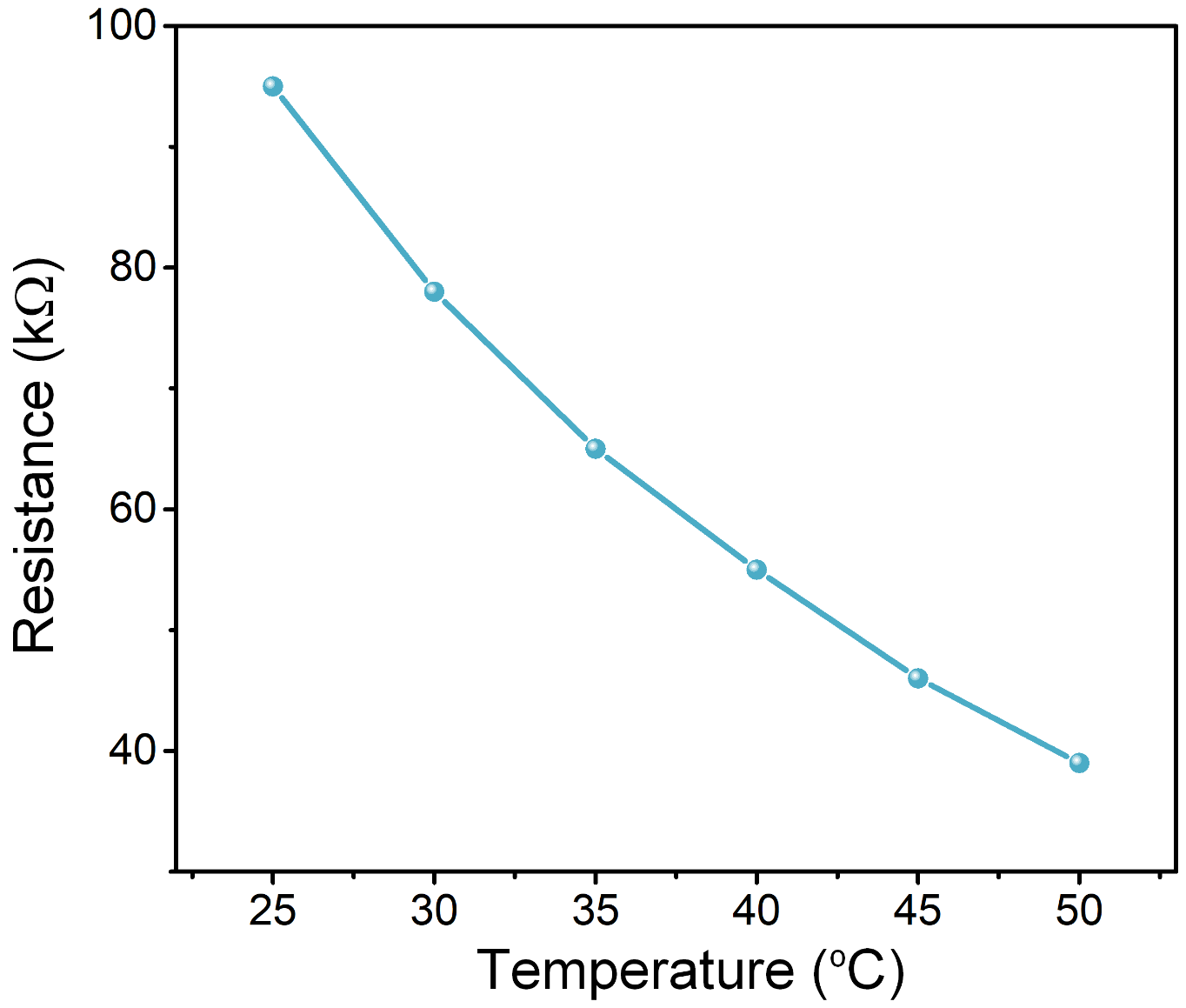


**Figure S4**. Temperature sensing property of the adopted temperature sensor driven by TEHNG. When the fabricated TEHNG was used to power the temperature sensor, it can work normally and continuously. As the temperature increased from 25 ℃ to 50 ℃, the resistance of the temperature sensor gradually decreased according to a certain rule.


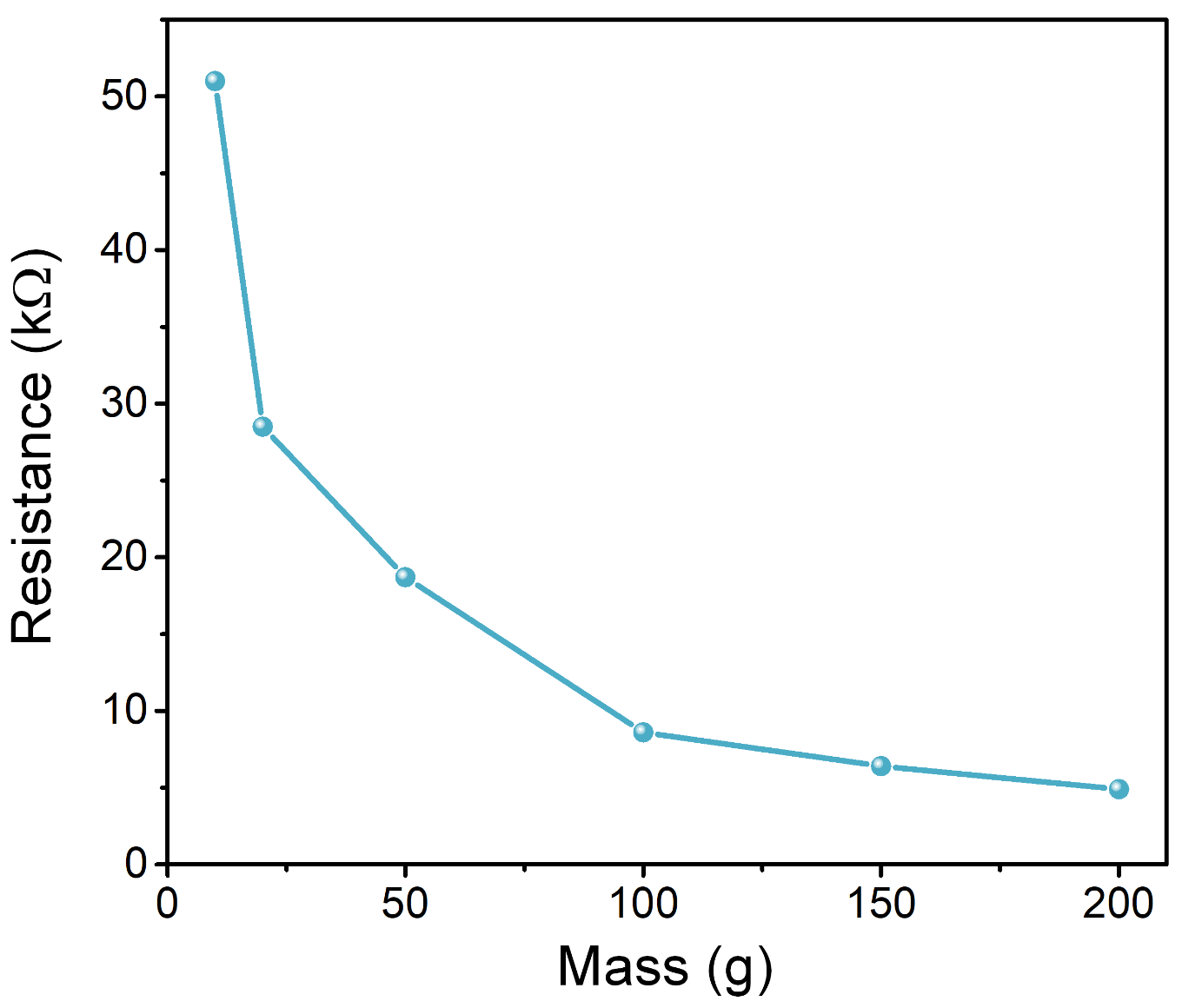


**Figure S5**. The quantitative relationship between the mass of weights and the resistance of the adopted pressure sensor driven by TEHNG. The pressure sensor can work normally and continuously when the fabricated TEHNG was used as the only power source. When the mass of weight increased from 10 g to 200 g, the resistance of the pressure sensor gradually decreased according to a certain rule. It is worth mentioning that the sensing range of the pressure sensor used is 0 g to 200g, therefore, when the mass of weight is close to 200 g, the resistance change of the pressure sensor is relatively small.


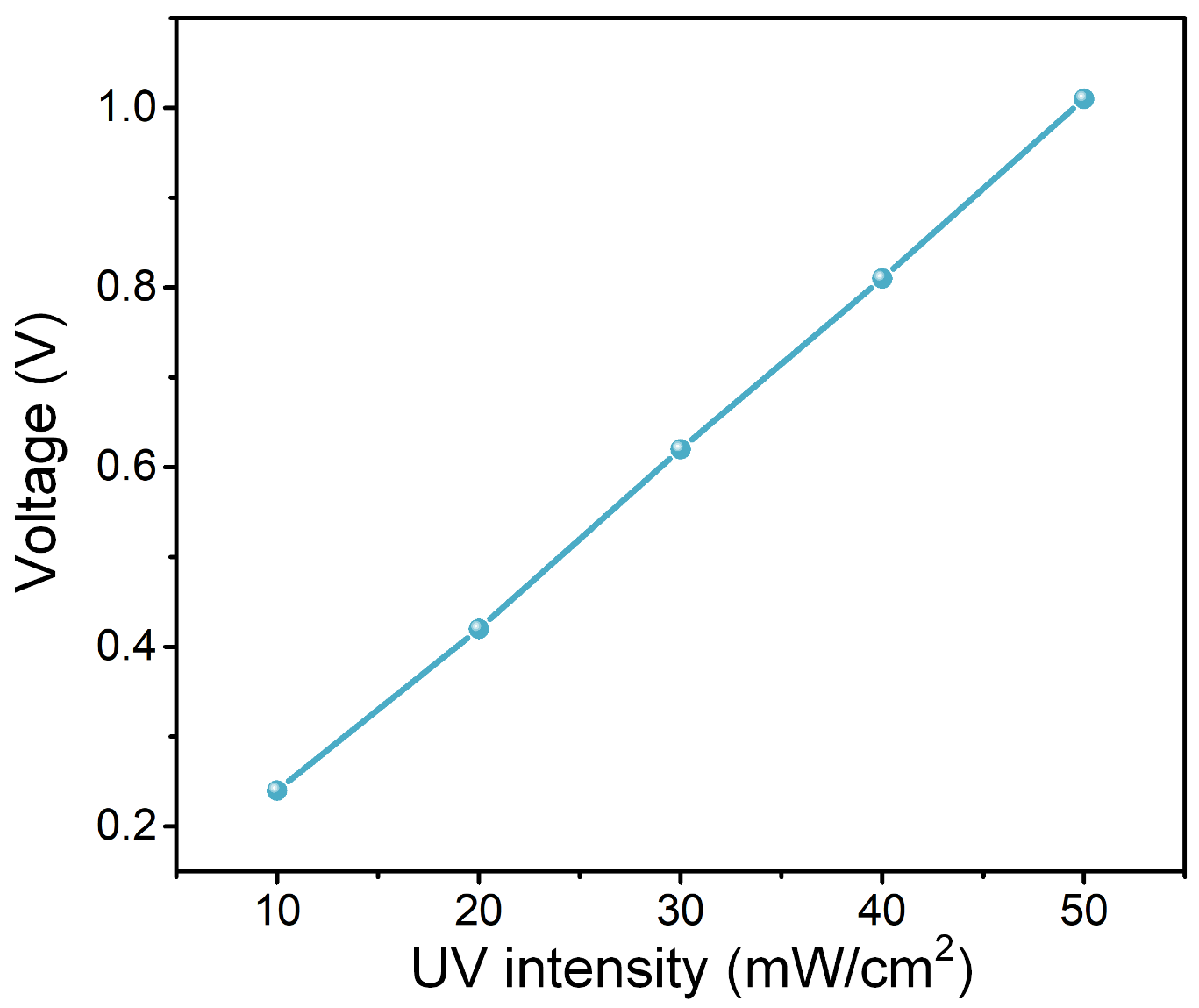


**Figure S6**. Output voltage of ultraviolet (UV) sensor as function of UV intensity from 10 mW/cm^2^ to 50 mW/cm^2^. The working power consumption of the UV sensor used in this work was 172 μW, and it still worked normally and continuously when it was powered by the fabricated TEHNG. The output voltage of the UV sensor increased linearly with the increase of the external UV intensity.


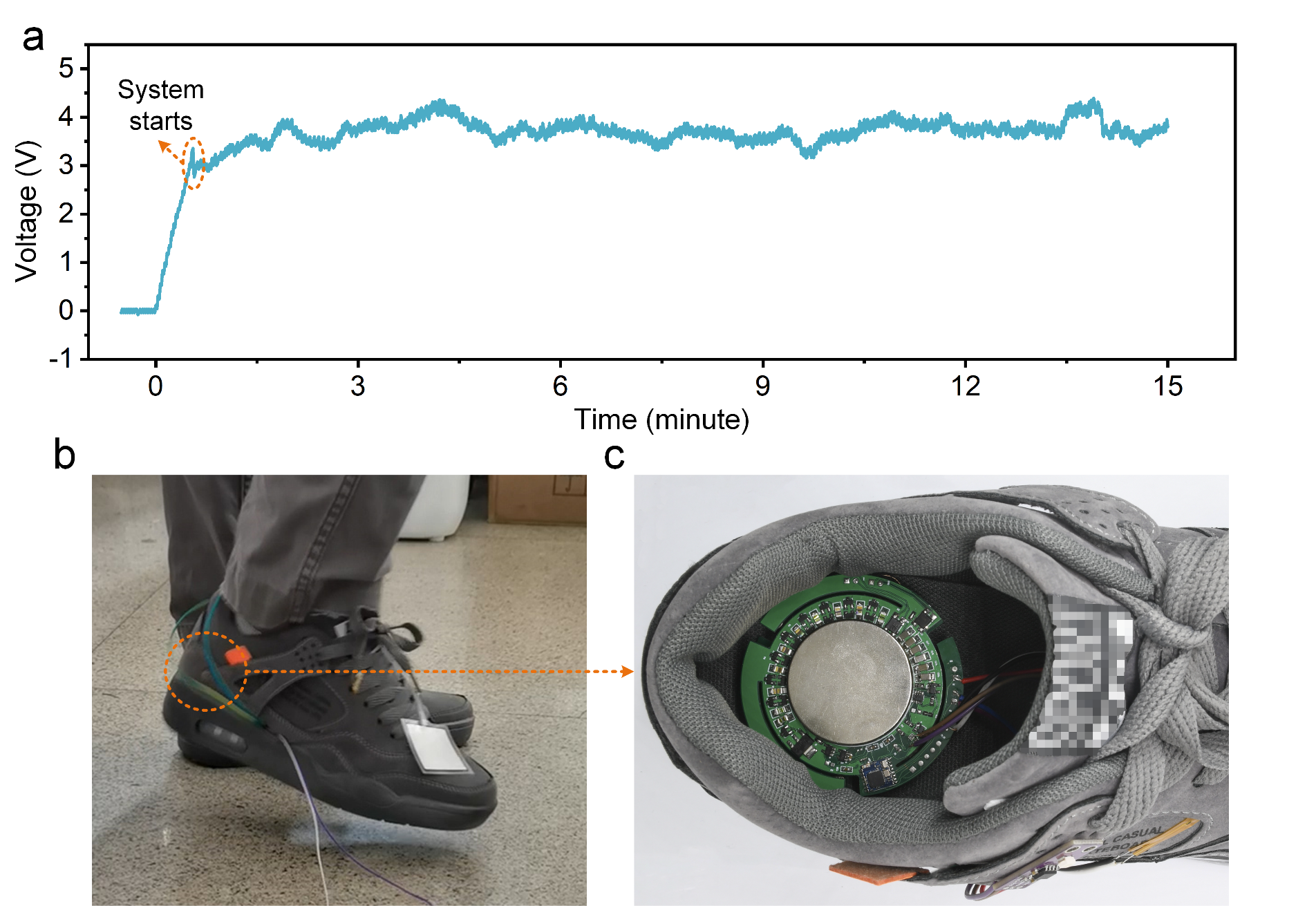


**Figure S7**. To verify the feasibility of the all-in-one self-powered multi-sensing microsystem and the stability of the device withstanding continuous pressure during normal human movement in practical applications, a 65 kg volunteer wore the microsystem inside his right shoe and kept walking in situ for 15 minutes. The operation frequency of one foot was ~1.1 Hz, and the corresponding output performance of the TEHNG was lower than that under 2 Hz foot stamping (Fig. 6). Therefore, we removed the UV sensor, and only temperature sensor and pressure sensor were connected to the self-powered microsystem. The data transmission frequency of the microsystem was set to 0.5 Hz. As a result, the self-powered multi-sensing microsystem can work continuously. (a) Voltage of the energy storage unit of the all-in-one self-powered multi-sensing microsystem. (b,c) The microsystem was worn inside a shoe. More details about the realistic test of a 15-minute walk are shown in **Video S2**.

TABLE SI

The energy transfer efficiency of the triboelectric part can be increased by 37.2% through PMM.

|  | **Original output** | **Transfer without PMM** | **Transfer with PMM** |
| --- | --- | --- | --- |
| **Average energy of a single pulse** | 1.729 μJ | 0.225 μJ | 0.868 μJ |
| **Efficiency** | - | 13.0% | 50.2% |

**Supplementary Videos**:

**Video S1**: The TEHNG was successfully demonstrated to drive the all-in-one multi-sensing microsystem to perceive environmental variables and send to cell phone.

**Video S2**: A realistic test of a 15-minute walk (~1.1 Hz for one foot) the feasibility of the all-in-one self-powered multi-sensing microsystem and the stability of the device withstanding continuous pressure during normal human movement in practical applications.

**References:**

[S1] Niu S. et al. Theoretical study of contact-mode triboelectric nanogenerators as an effective power source. *Energy Environ. Sci*., **6**, 3576(2013).
